# Has the Mammalian Blood Corpuscle a Nucleus?

**Published:** 1879-03

**Authors:** Wm. T. Belfield

**Affiliations:** Demonstrator of Physiology, Rush Medical College


### ARTICLE IV.

HAS THE MAMMALIAN BLOOD-CORPUSCLE A NUCLEUS? By WM. T. BELFIELD, M.D., Demonstrator of Physiology, Rush Medical College. Read before the Illinois State Microscopical Society.

The question of the existence of a nucleus in the red blood-corpuscle of mammals has for many years engaged the attention of physiologists. Three considerations suggest such an existence. First, that a nucleus is apparent in all blood-corpuscles not mammalian: second, that nuclei have been recognized in the blood-corpuscles of fœtal mammals; that a nucleus, or the suggestion thereof, can be readily demonstrated in almost all other anatomical elements of mammals. Hence, the deduction has been made that all elements of tissue require nuclei; therefore that the homology of tissues requires that blood corpuscles should also possess them. Prompted by such reasoning, many observers have sought patiently and persistently to demonstrate the existence of these hypothetical bodies, devising new means and methods when old ones had proved ineffectual. Several times success has been claimed, and not a few observers have asserted their ability to demonstrate these nuclei; but, as a recent contributor to this subject has said: "When their methods and work have been published, and others have followed their directions, errors have invariably been pointed out, and the so-called nucleus has been absorbed in 'optical delusions,' and changes brought about by the reagents used." It is somewhat remarkable that the writer of these words, Dr. Stowell, of Ann Arbor, in the same article claims to have demonstrated the nucleus by a new method; or more accurately speaking, claims to have repeated the work of Prof. Boettcher, with confirmatory results. The method is as follows: The corpuscles are bleached by means of a saturated solution of corrosive sublimate in 96 per cent. alcohol. Into 50 parts of this solution one of blood is rapidly diffused. In twenty-four hours the supernatant liquid is poured off and alcohol added. In twenty-four hours more this is replaced by distilled water. The corpuscles are then subjected to staining agents. By this method Prof. Boettcher claims to have seen nuclei in a small percentage of the corpuscles so treated.

Upon reading these observations it occurred to me that the asserted nucleus might be, in part at least, due to coagulation of albumen and extraction of water (which certainly occurs), whereby a central thickening might readily appear, and hence a difference of refraction and apparent deepening of color be observed.

It seemed that if simple bleaching alone were to be accomplished by the reagents used, the same results should follow bleaching by other methods. I therefore instituted a series of observations, whose results I place before you in this paper and on these slides.

Let me, as preliminary, define the nucleus; for much of the difference of opinion on this question results, it seems to me, from a failure to identify this body. According to Tyson, the nucleus is a mass of bioplasm in the interior of the cell, "more granular, or darker in hue, by transmitted light than the remainder of the cell." * * "This living matter exhibits the important property of being stained by weak solutions of various coloring matters, as carmine, aniline, etc.," * * * "and by these means its demonstration is rendered strikingly easy." If then, after exposure to such a solution, we observe in the interior of a cell a sharply-defined mass exhibiting unmistakable color, the remainder of the cell being colorless, we assert the existence of a nucleus; the absence of such differentiation demonstrating with equal force the absence of a nucleus.

With this idea in view, I procured specimens of fresh blood from man, the dog, rat and turtle; exposed the corpuscles to the action of various bleaching agents—chlorine, sulphurous acid, acetic acid, a freezing temperature; then, when the coloring matter had been removed, immersed them in weak solutions of aniline and carmine, and mounted them in distilled water. I was careful to produce, as nearly as possible, identical effects upon all the specimens, using the same solutions for the same periods upon them all. By each method nuclei were clearly demonstrated in the turtle's blood, but in no other specimen was there any differentiation of color. It is true that some mammalian corpuscles showed staining, but that staining was invariably uniform from center to periphery, proving conclusively the *absence* of a nucleus so far as carmine staining can prove anything.

These slides have been submitted to the scrutiny of several observers, among them Mr. A. F. Atwood, Drs. H. A. Johnson, Lester Curtis and Chr. Fenger. In every instance the result of the observation was announced before the observer was informed what specimen he was looking at, or why his observation was requested. Thus all possible bias and prejudice were avoided, although the names of these gentlemen are in themselves ample guaranty for the fidelity and accuracy of the observations. Therefore it was with great satisfaction that I heard from each an opinion coincident, in all its essentials, with my own. They observed, as I had in a few mammalian corpuscles, points of different refractive power from the rest of the corpuscle, but never any points of different staining.

The question at once arises—How are we to explain Boettcher's nuclei ? It would seem to me that they are due to the action of the reagents employed. Certainly both alcohol and corrosive sublimate coagulate albumen, and alcohol extracts water. That we can secure differences of refraction and even differences in depth of staining by reagents I have proved in this way : Mucus is structureless when taken from the mouth ; but when treated with Boettcher's fluid.it becomes fibrillated. Now the addition of carmine solution causes an uneven staining, the fibrillæ being especially colored. Surely no one would affirm that these fibrillæ consist of nuclear or germinal matter. Yet just as these fibrillæ differ from mucus as it exists in the mouth, so blood corpuscles, after treatment by Boœttcher's method, differ from blood-corpuscles in the vessels. This suspicion that Bœttcher's nuclei are artificial, receives support from recent discoveries as to the structure of nuclei. In the July number of the *Quarterly Journal of Microscopy*, Dr. Klein relates a series of observations, as a result of which he affirms the nucleus to consist of a fibrillar network imbedded in which is a ground substance; that this intra-nuclear network is continuous with a similar intra-cellular network; that nucleoli are merely thickenings and shrivelings of these fibrils. The natural shrivelling effect of alcohol might readily produce a pseudo-nucleus in a blood-corpuscle from condensation of this intra-cellular net work.

Now as to the argument that the homology of tissues demands the existence of a nucleus in the red corpuscle. Does this homology require nuclei in the enamel rods and superficial layers of the epidermis? Certainly they have never been demonstrated. Indeed a nucleus would be in these situations superfluous, for the functions of the nuclei are, as we understand it, growth, nutrition and reproduction. Since neither of these processes is required of enamel or superficial epithelium, nuclei are here unnecessary and therefore absent. Now the function of the mammalian blood corpuscle is only, so far as we know, the transportation of oxygen, a purely chemical process performed as well without as within the body. The hæmato-crystallin, like other ferrous salts, is capable of uniting with oxygen, and again, under other circumstances, of imparting that oxygen. Nutrition, growth and reproduction are, to the best of our knowledge, foreign to the matured corpuscle; hence a nucleus is unnecessary and we would, *a priori*, expect its absence. This proposition is advanced, not as an argument, but simply to show the fallacy of the argument based on homology.

It would be improper to conclude this article without reference to the work of the late Prof. Freer, of this city. By the use of reflected light, Prof. Freer demonstrated in many blood corpuscles a central thickening, but failed to prove any corresponding difference in chemical composition, vitality or function—that is, failed to prove the existence of a nucleus. Hence, while authorities universally admit his demonstration as to contour, they as universally restrict his demonstration to contour. If thickness constitutes a nucleus, then the red corpuscle has a nucleus all around its periphery as well as in its center.

